# Outcomes and Stability of Anterior Open Bite Treatment with Skeletal Anchorage in Non-Growing Patients and Adults Compared to the Results of Orthognathic Surgery Procedures: A Systematic Review

**DOI:** 10.3390/jcm10235682

**Published:** 2021-12-01

**Authors:** Piotr Malara, Susanne Bierbaum, Beata Malara

**Affiliations:** 1 Department of Maxillofacial Surgery for Children, Chorzow Hospital for Paediatrics and Oncology, 41-500 Chorzów, Poland; 2 Postgraduate Educational Centre of Dentistry DENTARIS, School of Medicine, Katowice Business School, 40-659 Katowice, Poland; 3 Max Bergmann Center of Biomaterials, Technische Universität Dresden, 01069 Dresden, Germany; s.bierbaum@med-college.de; 4 International Medical College, 48143 Münster, Germany; 5 Department of Facial Aesthetics and Cosmetology, School of Medicine, Katowice Business School, 40-659 Katowice, Poland; beata.malara@gwsh.pl

**Keywords:** anterior open bite, molar intrusion, skeletal anchorage, orthognathic surgery

## Abstract

The objective of this review is to evaluate, on the basis of the available literature, if anterior open bite (AOB) can be successfully treated with the intrusion of molar teeth using skeletal anchorage in non-growing patients and adults and if this treatment modality provides comparable results to those obtained by orthognathic surgery procedures. Methods: A systematic review of published data in major databases from 2000 to 2021 was performed. Results: In total, 92 articles were included in title and abstract screening, and only 16 articles (11 concerning AOB correction by molar intrusion with skeletal anchorage, and five considering AOB treatment by orthognathic surgical intervention) qualified for thorough data extraction and analysis. Conclusions: On the basis of this review, it seems to be possible to obtain successful results for AOB treatment in non-growing patients and adults by means of the intrusion of molar teeth with skeletal anchorage. However, due to the different methods of assessing treatment outcomes used by different authors, it is not possible to state conclusively whether the treatment of AOB by means of molar intrusion with skeletal anchorage provides long-term results that are comparable to orthognathic surgery procedures.

## 1. Introduction

Anterior open bite (AOB) is still one of the most difficult and demanding clinical problems. This malocclusion relies on a reduction in the vertical relationship between the incisal edges of the upper and lower incisors [1]. There are many etiological factors of AOB. These include genetic, skeletal, dental and functional factors; factors related to the morphology of soft tissues; and habits [2]. Accompanying symptoms of AOB include increased lower face height (LFH), short posterior face height (PFH), increased gonial and mandibular plane angles and higher maxillary molar dentoalveolar height [3]. AOB is very often associated with numerous dental abnormalities, including tooth crowding, followed by problems with chewing food and speech, as well as aesthetic defects. Moreover, AOB is accompanied by muscular and functional problems, such as incompetence of the lips and a convex facial profile [1]. The development of AOB is also associated with the existence of parafunctions, which include thumb sucking or tongue thrust [4].

The development of orthodontics has provided many varieties of treatment for both dental and skeletal forms of AOB. The proposed treatment methods include both functional appliances and fixed appliances. Orthognathic surgical procedures also play an important role in the treatment [1].

In children, it is relatively simple to control facial growth through a variety of functional therapies. In this way, blocking the growth of the lateral parts of the alveolar process and provoking the growth of the dentoalveolar complex in the anterior region provide treatment options for AOB [5]. The treatment of AOB in non-growing patients and adults is much more difficult due to the inability to influence the skeletal development of the facial part of the skull, as well as the high susceptibility to relapse after orthodontic intervention in the dentoalveolar complex.

Traditionally, in patients with accomplished musculoskeletal development, the gold standard of treatment of AOB is orthognathic surgery [6]. The surgical treatment of AOB includes solely LeFort I osteotomy (LIO) or in conjunction with bilateral sagittal split osteotomy (BSSO) procedures performed on the mandible [2,7,8,9]. Orthognathic surgery modalities offer the best possible three-dimensional correction of both the facial skeleton and the dentoalveolar complex. It should be emphasized that the diverse range of procedures on the maxilla and the mandible that are collectively described as orthognathic surgery procedures are recognized to be safe surgical interventions [10,11]. However, there are a number of unusual and rare complications of orthognathic surgery that a surgeon should stay vigilant about. Those risks are dependent on the technique used and the skills of the surgeon [12]. It must be taken into account that there is a need to use intermaxillary fixation for 4–8 weeks after surgery and to stay in hospital for a few days after surgery [13], which can be inconvenient for the patient. The economic aspect of orthognathic treatment is also important, including not only the costs of the surgery itself, but also the necessity to refrain from social and professional life for 6–8 weeks after the surgery. Due to the information presented above, the acceptance of surgical and orthodontic treatment plans among patients is relatively low [14].

The development of non-surgical treatments for AOB increases the availability of treatment to patients. The desired effect of non-surgical AOB treatment was the extrusion of the incisors, which led to an increase in the anterior overbite, but also to the unsightly elongation of the anterior teeth [15]. It should also be mentioned that extrusion is a much less stable tooth movement option than intrusion. Molar intrusion in the treatment of AOB has been a suggested treatment option for many years. However, only the development of skeletal anchorage techniques enabled the predictable and safe implementation of tooth intrusion into clinical practice [16].

Among the methods of AOB treatment, the intrusion of molars with the use of temporary anchorage devices (TADs) has a unique value. Mini-implants, mini-screws or mini-plates can be used as temporary skeletal anchorage [17]. The objective of this treatment option is to intrude the molar teeth by exerting a force between the temporary anchorage placed on the bone and the orthodontic appliance. This procedure allows a positive overbite to be achieved on the incisors by the intrusion of molar teeth followed by auto-rotation of the mandible [18].

The aim of orthodontic and surgical-orthodontic treatment is to correct malocclusion and achieve stable long-term treatment results. AOB is among the dentoalveolar and skeletal problems characterized by a high relapse rate [19]. Therefore, it is important to critically evaluate the newly introduced methods of treatment in terms of the stability of the achieved treatment effects. The assessment can be performed on the basis of repeatable measurements reflecting dentoalveolar and skeletal components of AOB. Among these measurements, the most important are the measurement of overbite; anterior facial height (AFH) which represent the main treatment outcomes; and position of the mandible in relation to the palatal plane (PP), Frankfort horizontal plane (FH), sella–nasion (SN) line or true horizontal line (THL) representing secondary outcomes of AOB treatment.

Taking into account the difficulties and complexity of AOB treatment, the multifactorial etiology of this malocclusion with both skeletal and dental components, the severity of both orthodontic and surgical treatment of this condition and the necessity of obtaining long-term results of the treatment, it seems to be interesting from the clinical point of view if the less invasive approach of AOB treatment using skeletal anchorage provides at least the same outcomes as traditionally applied orthognathic surgery procedures.

The objective of the study was to systematically review if AOB can be successfully treated with the intrusion of molar teeth using skeletal anchorage in non-growing patients and adults and if this treatment modality provides comparable results to those obtained by orthognathic surgery corrections. To this aim, the linear change in overbite on the incisors and the angular measurements of mandibular autorotation that follow either the intrusion of molar teeth with skeletal anchorage or the orthognathic surgery procedures were analyzed.

## 2. Materials and Methods

This study was carried out according to the PRISMA statement for reporting systematic reviews of health sciences [20]. The strategy was based on an electronic search of articles published from January 2000 until December 2021 in the following databases: PubMed, Web of Science, Google Scholar, Embase, Ovid and Scopus. The Medical Subject Headings (MeSH) used for the search are shown in Table 1.

Individual search terms and cross-linked search terms were processed in the databases.

### 2.1. PICOS Framework

Population: adults and non-growing patients with no regard to gender with AOB treated by molar intrusion with skeletal anchorage (Q1) or by orthognathic surgery procedures, including LIO with or without BSSO mandibular surgery (Q2). “Adults” were defined as over 18 years of age and “non-growing” as patients with completed craniofacial growth. Patients were considered to have AOB malocclusion if they had negative values of overbite measured on the incisal edges of the upper and lower central incisors (U1 and U2).

Intervention Q1: the intrusion of permanent molar teeth with skeletal anchorage. Skeletal anchorage could have been achieved through either mini-plates on the zygomatic buttress fixed with bone screws or temporary anchorage devices in the form of mini-screws or mini-implants anchored in the area of the molars from the vestibular side. Connection between the orthodontic appliance and the anchoring elements consisted of power chains or NiTi coil springs; additional elements such as acrylic splints or transpalatal arch bars were allowed.

Intervention Q2: the orthognathic surgery procedures including LIO with or without BSSO mandibular surgery.

Comparator: Studies comparing linear measurements of the overbite on the incisors and the angular measurements of the position of the mandible against the palatal plane or the skull base taken pre-treatment (T1), post-treatment (T2) and at least one year into retention (T3).

Outcome (Q1 and Q2): the primary outcome was to achieve the positive values of overbite measured between the incisal edges of the upper central incisor (U1) and the lower central incisor (L1) followed by a reduction in linear measurements of AFH or LFH, and the secondary outcome was to obtain the auto-rotation of the mandible expressed by the angular change in the position of the mandible in relation to PP, FH, SN line or THL determined by cephalometric analysis performed on the lateral skull telecephalograms.

Study design: it was planned to search for randomized and non-randomized clinical trials, cohort studies and case series with over 5 cases included.

### 2.2. Review Questions

1. Is it possible to manage AOB in non-growing patients and adults by means of the intrusion of molar teeth with skeletal anchorage?
2. What are the outcomes of AOB treatment by molar teeth intrusion with skeletal anchorage or by orthognathic surgery rated by comparisons of the measurements taken pre-treatment (T1) and post-treatment (T2)?
3. Does the treatment of AOB by means of molar intrusion with skeletal anchorage provide the same long-term results as the orthognathic surgery correction assessed by comparisons of the measurements taken post-treatment (T2) and at least one year into retention (T3)?

### 2.3. Inclusion Criteria

- Human studies;
- Articles concerning adults (over 18 years of age) and non-growing individuals;
- Randomized and non-randomized clinical trials;
- Cohort studies
- Cases series studies with at least 5 cases included;
- Articles assessing long-term results of the treatment.

### 2.4. Exclusion Criteria

- Case reports and cases series studies with less than 5 cases included;
- Animal studies;
- Review articles;
- Articles with a follow-up period of less than 12 months

### 2.5. Article Selection

Articles obtained by the electronic search were screened by titles and abstracts by three reviewers independently (P.M., S.B. and B.M.). As it is impossible to rely on the abstracts only, no attempt was made to find all the data regarding changes in overbite and mandibular rotation in the abstracts immediately. Therefore, even articles on AOB treatment by molar intrusion with skeletal anchorage or orthognathic surgery whose abstracts did not mention results of mandibular autorotation qualified for the next stage of the selection process. An agreement on the eligibility of articles for this systematic review was reached among assessors via discussion.

### 2.6. Data Extraction

Two authors (P.M. and B.M.) carried out the data extraction process in duplicate. The following items were collected: type of intervention, study design, number of patients involved, mean age of patients, mean time of active treatment, data characterizing overbite and mandibular position before and after treatment, maximum follow-up time, the change in measurements characterizing overbite and the position of the mandible. In the case of any disagreement during data extraction, a consensus was achieved by discussion with the third assessor (S.B.).

### 2.7. Assessment of the Risk of Bias

The risk of bias in the articles included in this review was assessed by two authors (PM and BM) in accordance with the recommendations described by Ma et al. [21]. The non-randomized clinical trials were assessed using the methodological index for non-randomized trials tool (MINOR) (Table 2), the cohort studies by the National Institute of Health (NIH) quality assessment tool for observational cohort studies (Table 3) and the cases series by the Institute of Health Economics (IHE) Appraisal Tool (Table 4) developed by Moga et al. [22] (Table 2). The items scored 0 if not reported, 1 if partially reported and 2 if fully reported. The overall risk of bias was calculated as the arithmetic mean of the scores and expressed on the scale from 0 to 2 where 0 represented the highest risk of bias. In the case of a disagreement, a consensus was reached by discussion. The consensus rating obtained by discussion was compared with the rating given by each reviewer. The consistency of scoring among the reviewers was assessed by interrater reliability analysis using the Kappa statistic. The results showed a substantial level of agreement between the reviewers (Kappa = 0.788).

### 2.8. Assessment of the Strength of Evidence for the Evaluated Outcomes

The strength of evidence for the main evaluated outcomes was assessed by two authors (P.M. and B.M.) using the grades of recommendation, assessment, development and evaluation (GRADE) approach [23]. The assessment was performed for the outcomes for which pooled results had been obtained. The overall quality of the studies was rated as “high”, “moderate” or “low”. The “importance” of the studies was determined by consensus between the two authors (P.M. and B.M.) and reported as “not important”, “important” or “critical”. The results of the strength of evidence for the evaluated outcomes are shown in Table 5.

### 2.9. Synthesis of the Extracted Data

We planned to perform a meta-analysis, but due to the high level of heterogeneity of the data, only a systematic review and qualitative analysis of the data were carried out.

## 3. Results

### 3.1. Characteristics of the Included Studies

**Figure 1:**
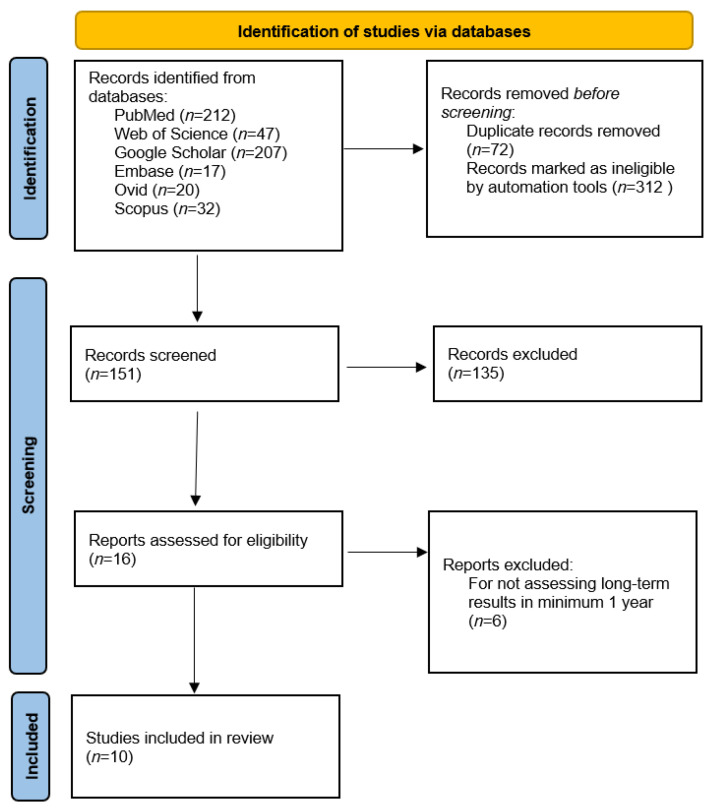
Flow diagram of the literature search.

Among 535 articles obtained as a result of an electronic search in the databases, 312 were excluded by automation tools as ineligible, and 72 were duplicate articles. Furthermore, 151 articles qualified for title and abstract screening, and only 16 of the articles were eligible for full-text reading, as the others did not meet the inclusion criteria. Out of 16 full-text articles, six additional articles were excluded as they did not assess long-term treatment results for a minimum of 1 year after treatment, enabling the stability of the achieved results to be assessed [18,24,25,26,27,28]. Out of 10 eligible articles, five focused on AOB correction by molar intrusion with skeletal anchorage and five considered AOB treatment by orthognathic surgical intervention including LIO with or without BSSO mandibular surgery. The process of article selection is shown in Figure 1. The calculated overall risk of bias for the group of articles regarding molar intrusion with skeletal anchorage and orthognathic surgery was 1.31 and 1.13, respectively. These results indicate a moderate level of the risk of bias in the articles regarding both types of intervention. It must be emphasized that the authors decided to include four articles with a very limited number of cases in this systematic review [2,7,8,9].

Two articles on AOB treatment with molar intrusion provided information on treatment outcomes 1 year after treatment completion [7,8]; one article—2 years after treatment completion [29]; and two articles—up to 3 years after the end of treatment [2,30]. In the group of articles evaluating the results of AOB treatment with orthognathic surgery, two articles assessed long-term results 1 year after surgery [31,32]; two articles—up to 3 years after surgery [6,33]; and one article—up to 15 years after surgery [9]. One article did not report the results of treatment immediately after surgery [33].

In treating AOB with skeletal anchorage, the authors used different treatment techniques and, therefore, different TADs. The primary method of obtaining skeletal anchorage for molar intrusion was the use of mini-plates placed on the zygomatic buttress and fixed with bone screws [7,30]. Other authors used temporary anchorage devices in the form of mini-screws or mini-implants anchored in the area of the molars from the vestibular side [2,8,29].

In most articles, power chains or NiTi coil springs reaching directly to the fixed orthodontic appliances were attached to the anchoring elements. In only two cases, the authors used additional elements placed on the teeth in the form of acrylic splints [29]. Additionally, in one article, the authors also used transpalatal arch bars [2].

In the articles assessing the results of AOB treatment using orthognathic surgery, two papers were only concerned with bimaxillary procedures consisting of LIO on the maxilla and BSSO on the mandible [9,32]. Teittinen et al. [6] and Proffit et al. [33] separately analyzed the treatment outcomes of patients who underwent only maxillary surgery using LIO or bimaxillary surgery. Swinnen et al. [31] also divided patients into two groups. The first group was treated with maxillary intrusion, and the second group, with maxillary extrusion with a maxillary or bimaxillary approach in each group. The characteristics of the studies included in this review are shown in Table 6.

In the articles included in this review, the authors used cephalometric analysis performed on lateral skull telecephalograms to evaluate the results of AOB treatment. Since many different analyses and parameters are used in cephalometric analysis, in the articles included in this review, the same treatment outcomes were often determined using different parameters. The measurement that was used in all eligible articles was overbite, understood as the distance between the incisal edges of the upper central incisor (U1) and the lower central incisor (L1) perpendicular to the horizontal reference line (HRL). On the other hand, different authors used different parameters indicating a reduction in facial height or mandibular autorotation as a result of treatment. In order to determine the change in facial height as a result of treatment, three articles used the LFH parameter, understood as the distance from the anterior nasal spine (ANS) to the menton (Me) [7,8,29], while Baek et al. [2] used the AFH parameter defined as the distance from the nasion (N) to the menton (Me). In one article on the surgical treatment of AOB, the authors used a linear measurement of N-Me or ANS-Me instead of AFH and LFH [31].

An even greater variety of terms related to the different parameters used by the authors relating to the autorotation of the mandible as a result of AOB treatment. Most often, the authors defined MP as a line passing through the cephalometric points gonion (Go) and gnathion (Gn) or Go and menton (Me) and the SN as a line passing through the points sella (S) and nasion (N) [2,6,8,9,29,30,31,32]. In one article, the authors used the angle created by MP to FHP, defined as the plane passing through the highest points of external auditory canals and the lowest point on the lower margin of the left orbit [7].

In articles using molar intrusion for the treatment of AOB, the authors also used linear measurements of the distance between the mesial buccal cusp of the first upper molar and the PP [2,7,8,29,30].

In order to avoid misunderstandings when using specific cephalometric parameters, the measurements related to the subject of this review from selected articles are presented in Table 4, where their definitions are also provided. The parameters used by the authors in the articles that qualified for this review are presented in Table 7.

### 3.2. Results of AOB Treatment Assessed by Achieving Positive Overbite on the Incisors and Other Parameters of AFH

Regardless of the treatment option chosen, the primary outcome of AOB treatment is a positive overbite on incisors. Therefore, all articles included in this review used the overbite parameter. Changing the value from negative to positive indicated the correct treatment outcome on incisors, regardless of whether the treatment was based on molar intrusion TADs or as a result of maxillary or bimaxillary orthognathic surgery.

In all cases, AOB treatment resulted in a reduction in the measurements of AFH, understood as the linear distance between N and Me, and a decrease in LFH, defined as the linear distance between the anterior nasal spine (ANS) and Me or ANS-Me distance.

**Figure 2:**
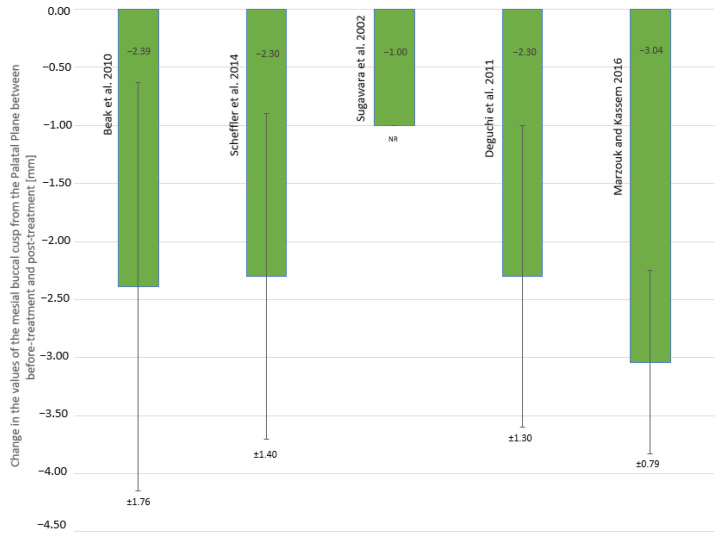
Change in the values of the distance of the mesial buccal cusp of the first upper molar from the palatal plane (mm); NR—not reported.

The values of overbite measured before and after AOB treatment using molar intrusion with skeletal anchorage alongside the calculated change in the vertical relationship between the incisal edges of U1 and L1 are summarized in Table 8, while the values of change in the distance of the mesial buccal cusp from PP are shown in Figure 2.

In articles on the treatment of AOB by molar intrusion, the primary pre-treatment overbite values ranged from −1.2 ± 1.7 mm [29] to −4.7 ± 2.3 mm [30]. The overbite achieved after treatment ranged from 1.0 mm [29] to 2.18 ± 0.48 mm [30]. Marzouk and Kassem [30] showed the highest value of the difference between overbite before and after treatment, amounting to 6.93 ± 1.99 mm.

A prerequisite for obtaining a correct overbite on incisors during AOB treatment is the achievement of molar intrusion. In the articles qualified for the review, the linear range of intrusion of the first upper molars, expressed as the difference in the distance between the mesial buccal cusp and PP, ranged from −1.0 mm [7] to −3.04 ± 0.79 mm [30].

The values of AFH and LFH before and after treatment and the mean change in these values are summarized in Table 9. In the articles qualified for this review, changes in LFH as a result of molar intrusion ranged from −1.50 mm [7] to −2.60 ± 2.50 mm [8]. Changes in facial height (Table 8), however, were not correlated with the range of the first upper molar intrusion (Figure 2) or the change in overbite (Table 8).

The results of AOB treatment using orthognathic surgery techniques assessed on the basis of overbite are summarized in Table 10. The greatest overbite change was found in patients subjected to bimaxillary surgery in the studies by Ding et al. [9] (3.8 mm). It should be noted that in these studies there was also the highest negative overbite value on central incisors before surgery, amounting to -3.2 mm. After bimaxillary surgical treatment, the highest overbite of 1.3 ± 1.1 mm was obtained in the article by Fisher et al. [32]. In studies analyzing the results of AOB treatment with the division into maxillary and bimaxillary surgery, a greater value of overbite change was obtained in the group of patients who underwent maxillary surgery (3.78 mm) than in the group who underwent bimaxillary surgery (3.17 mm) [6]. At the same time, in the same studies, the highest value of overbite after surgery was obtained in the group of patients treated with LIO only (1.23 ± 1.05 mm).

Only in one article did the authors analyze changes in the parameters characterizing facial height as a result of AOB treatment using orthognathic surgery [31]. In this article, as a result of orthognathic surgery with maxillary intrusion, a decrease of 5.5 mm in the values of N-Me and ANS-Me was obtained, while in the group of patients treated with maxillary extrusion, the decrease in these values was only 0.8 mm. It should be noted that the values of N-Me and ANS-Me in both groups changed by the same value, which indicates that the decrease in the ANS-Me distance was responsible for the change in AFH (Table 11).

### 3.3. The Effects of AOB Treatment Assessed by Achieving Positive Overbite on the Incisors and Other Parameters of AFH

In the treatment of AOB, mandibular autorotation occurs with the overbite change following the intrusion of molars (Table 8) or as a result of orthognathic surgery (Table 10). In the cephalometric analysis on lateral cephalograms, mandibular counterclockwise rotation (CCR) should be found, which is expressed by negative values of the change in angular measurements characterizing the angle between the plane of the mandible and the higher horizontal lines. These measurements include SN-GoMe, SN-GoGn, PP-MP, FMA, MP-FH and MP-SN (Table 7).

It should be emphasized that the changes in angular measurements characterizing mandibular autorotation shown in Table 11 were not related to the achieved overbite changes (Table 8). However, an obvious relationship was noticed between the changes in the values of angular measurements characterizing mandibular autorotation and measurements characterizing facial height (Table 9).

In selected articles on AOB treatment with orthognathic surgery techniques, the greatest negative change in MP-SN was found in the studies by Fisher et al. [32] and Teittinen et al. [6] in groups of patients subjected to bimaxillary surgery, which were equal to −4.0 ± 3.1 and −4.6 (SD not reported), respectively (Table 13). These values were not correlated with significant changes in overbite and facial height.

Ding et al. [9] showed that in a group of patients with AOB treated with bimaxillary orthognathic surgery, the lowest negative changes in MP-SN were −1.3 degrees, accompanied by the largest changes in MP-PP of −6.1 degrees (Table 12). In this way, these authors obtained significant changes in the mean overbite of 3.8 mm (Table 10). The highest values of changes in angular MP-PP measurements were obtained by Teittinen et al. [6] in a group of patients with AOB treated with bimaxillary orthognathic surgery, which was −7.33 degrees. In this case, the mean change in the angular values of MP-SN measurements was only −4.6 degrees (Table 13), with an average overbite change of 3.17 mm (Table 10).

The stability of the obtained treatment results can be evaluated on the basis of repeated measurements of selected parameters in a cephalometric analysis over a longer time period. The long-term results of AOB treatment by molar intrusion, assessed on the basis of overbite; facial height; and cephalometric measurements of PP-SN, MP-SN and MP-PP, are shown in Table 14.

With the subsequent decrease in overbite and increasing facial height and molar extrusion, distorotation of the mandible occurs, as evidenced by the increasing values of SN-GoMe, SN-GoGn, MP-SN and MP-FH. These changes are small, and after 1 year of follow-up, they range from 0.29 degrees of SN-GoMe [2] to 1.6 degrees of MP-SN [8]. Only Scheffler et al. [29] found no change in SN-GoGn angle one year after the end of treatment. Three years after the end of treatment, distorotation of the mandible was found from 0.2 degrees of MP-SN [26] to 0.57 degrees of SN-GoMe [2].

Long-term results of the surgical treatment of AOB after a follow-up period of 1 to 15 years after surgery, including overbite and angular values of the position of MP to PP and MP and PP to the skull base, are shown in Table 15.

Following bimaxillary surgery, Ding et al. [9] found an increase in overbite of 0.9 mm after 15 years, and Swinnen et al. [31], 0.5 to 0.6 mm 1 year after surgery. On the other hand, other authors found a decrease in overbite over long periods of time after bimaxillary surgeries, amounting to −0.5 mm after 1 year [32], −0.25 mm after 3 years [33] and −0.25 mm after 3.5 years [6]. However, after LIO, there was always an increase in overbite a long time after surgery, ranging from 0.02 mm after 3 years [33] to 0.62 mm after 3.5 years [6].

After surgical-orthodontic treatment, in the long-term follow-up, counterclockwise (CCW) rotation of PP, clockwise (CW) rotation of MP and an increase in MP-PP angle occur, which indicates a tendency to relapse of the skeletal AOB component. The highest value of the increase in the MP-PP angle amounting to 4.96 degrees was found by Teittinen et al. [6] after 3.5 years of bimaxillary surgery follow-up.

In general, after bimaxillary surgery, over long periods of follow-up, there was always a greater distorotation of the mandible, ranging from 0.9 degrees of MP-SN after 15 years [9] to 3.77 degrees of MP-SN after 3.5 years [6]. Furthermore, 3.5 years after LIO distorotation of the mandible, 1.67 degrees of MP-SN was found [6].

## 4. Discussion

With the advent of skeletal anchorage methods, molar intrusion has become an effective alternative to surgical-orthodontic complex treatment of AOB [7]. Orthodontic molar intrusion is a method that does not require patient cooperation, and the placement of TADs is a much less invasive procedure than orthognathic surgery [30].

It is emphasized that both orthodontic and surgical-orthodontic treatment modalities are associated with a high relapse rate [33]. Historically, many methods of purely orthodontic treatment of AOB have been used. In the case of orthodontic treatments of AOB with extractions, the relapse rate could be as high as 25.8% [19]. An even higher relapse rate of up to 38.1% was found in cases of traditional non-extraction AOB treatment [34].

It must be emphasized that four of the articles included in this review were examples of research works based on a very limited number of cases [2,7,8,9]. It must be taken into account that for a high-quality systematic review, only well-designed prospective randomized clinical trials should be included, which would generate a firm evidence-based assessment of several treatment modalities [35]. However, it is not uncommon, especially in systematic reviews on new surgical interventions, that due to the limited number of articles on the specific topic, authors include non-randomized clinical trials, retrospective studies and even case series [36]. It is clearly an exception to the rule and to conduct this, several bias-related tools must be applied, which was carried out in this systematic review [35].

In all articles on AOB treatment with molar intrusion, a positive overbite was achieved as a result of shortening the distance between the mesial buccal cusp of the first molar and PP. Another consequence of molar intrusion is the shortening of AFH affecting the facial appearance. The consequence of this phenomenon is also the CCW rotation of the mandible. It should be noted that all articles included in this review, with the exception of the articles on the surgical treatment of AOB, used skeletal anchorage as the point of application of the force triggering molar intrusion. Importantly, however, the differences in this procedure concerned both the positioning of the TADs on the craniofacial skeleton and the use of other orthopedic components during the treatment. The use of mini-plates fixed with bone screws at a zygomatic buttress significantly accelerated the pace of molar intrusion, made it possible to use greater orthodontic forces and reduced the risk of loosening of the anchoring elements [7,30].

Reductions in AFH or LFH due to molar intrusion in the treatment of AOB were reported in all the research works included in this review. It should be noticed that changes in AFH or LFH were not correlated with an increase in overbite.

In all articles included in this review on the treatment of AOB by molar intrusion, the increase in overbite and decrease in AFH were accompanied by CCW rotation of the mandible. This was the result of a change in the occlusal plane of the maxilla and the subsequent anterorotation of the mandible. The authors emphasized that CCW rotation of the mandible contributed to the improvement of facial aesthetics [7,30]. It should be noted that prior to the introduction of TADs, traditional orthodontic treatment did not allow for a change in the occlusal plane of the maxilla through the intrusion of the molars, and thus did not allow for CCW rotation of the mandible [34]. Moreover, extrusion of incisors, which was possible without the use of TADs, was at risk of a much higher rate of relapse [19].

The information cited above and the results of the research included in this review allow us to conclude that it is possible to obtain successful results of AOB treatment in non-growing patients and adults by means of the intrusion of molar teeth with skeletal anchorage and a positive overbite on incisors, followed by decreases in AFH and CCW rotation of the mandible.

Another problem addressed in this review is determining the linear change in overbite measured on the incisors, linear change in AFH and the angular change in mandibular autorotation in non-growing patients and adults with AOB treated with molar teeth intrusion with skeletal anchorage compared to individuals subjected to orthognathic surgery. When comparing the results of AOB treatment via molar intrusion with the results of surgical treatment, the basic parameter of treatment effectiveness, which achieves a positive overbite on the incisors, should be taken into account. As a result of molar intrusion, in the articles qualified for this review, a greater range of overbite change as a result of treatment with molar intrusion was obtained, ranging from 2.2 ± 1.60 mm [29] to 6.93 ± 1.99 mm [30]. In the case of surgical AOB treatment, the overbite change ranged from 1.9 [31] to 3.8 mm [9]. It should be noted, however, that in the long-term follow-up, there may be a further increase in overbite on central incisors, even up to 0.9 mm after 15 years [9].

In the case of surgical-orthodontic treatment of AOB, significant differences in the change in AFH should be taken into account due to the different methods of surgical treatment used. In the selected group of articles qualified for this review, only one study assessed changes in AFH after surgical-orthodontic treatment of AOB. The changes in AFH ranged from −0.8 mm in the case of surgical procedures with maxillary extrusion to as high as −5.5 mm in the case of surgical procedures with maxillary intrusion [31]. It appears that surgical methods of AOB could make major changes in AFH compared to AOB treated with molar teeth intrusion. It should be emphasized that, as a result of the surgical treatment of AOB with the use of BSSO, an increase in PFH can be achieved, which, according to some authors, is associated with a higher risk of relapse of AOB [33,37].

One of the effects of AOB treatment, regardless of the treatment method used, is the angular change in the position of the mandible. In the case of AOB treatment by molar intrusion, the position of the mandible changes as a result of mandibular autorotation. The same mechanism is the reason for changing the position of the mandible as a result of LIO orthognathic surgery. Of the studies on AOB treatment with surgical-orthodontic methods qualified for this review, only one assessed the influence of LIO in the treatment of AOB on the MP-SN angle value [6]. The change in this value was −4.6 degrees, which suggests that it could be possible that surgical intervention allows greater angular values of mandibular autorotation than molar intrusion to be obtained, but this statement is subject to great uncertainty due to the different measurements used in different research papers.

Orthognathic surgery offers the possibility of changing the angular values of the position of not only the mandible but also the maxilla. The result of these procedures may be a positive change in the angle of PP to MP with slight negative changes in the angle of MP to the skull base. Therefore, in the group of patients with AOB treated with bimaxillary orthognathic surgery, slight negative changes in MP-SN of −1.3 degrees were obtained, accompanied by the largest changes of −6.1 degrees in MP-PP, resulting in significant changes of 3.8 mm in the mean overbite [9].

Based on the above considerations, it should be concluded that the treatment of AOB by molar intrusion allows a larger positive overbite on incisors than surgical treatment immediately after surgery, but a smaller range of changes in AFH or LFH, to be obtained. Molar intrusion in AOB treatment causes a greater CCW rotation of the mandible than AOB treatment with BSSO or bimaxillary surgery, but less than with LIO alone.

The aim of orthodontic or surgical-orthodontic treatment of malocclusion is always to correct the malocclusion and maintain stable treatment results over a long period of time. Therefore, another problem raised in this review was determining whether the treatment of AOB by means of molar intrusion with skeletal anchorage provides the same long-term results as the orthognathic surgery correction measured by the linear decrease in overbite and angular increase in mandibular distorotation with time in non-growing patients and adults.

The results of AOB treatment with molar intrusion using skeletal anchorage indicate that, one year after the end of treatment, molars are always susceptible to re-extrusion from 10% [7] to as much as 21.74% [29]. The consequence of this process is the reduction in overbite with time, which ranges from −0.8 [8] to −0.99 mm [2] within one year after the end of treatment and continues for many years. Among the articles selected for this review on the stability of the results of AOB treatment with molar intrusion, the longest follow-up period was 3 years, and after this time, the overbite decreased from −0.2 [30] to −0.44 mm [2]. Distorotation of the mandible also occurs after AOB treatment with molar intrusion, as evidenced by increasing values of SN-GoMe, SN-GoGn, MP-SN and MP-FH. These changes are small, and after 1 year of follow-up, they range from 0.29 degrees of SN-GoMe [2] to 1.6 degrees of MP-SN [8]. Only Scheffler et al. [29] found no change in the SN-GoGn angle one year after the end of treatment. Three years after the end of treatment, distorotation of the mandible was found from 0.2 degrees of MP-SN [26] to 0.57 degrees of SN-GoMe [2].

In the literature on the long-term outcomes of AOB surgery, the authors emphasize that the post-operative stability of AOB treatment depends on the type of surgery. Maxillary surgery involving LIO is considered more stable than BSSO or bimaxillary surgery as the activity of the masticatory muscles does not affect the maxillary procedures [33,37]. Data on the stability of overbite following surgical treatment of overbite are not conclusive. After orthognathic surgery, in long-term observation periods, there is a CW rotation of MP and an increase in the MP-PP angle, which indicates a tendency of the skeletal AOB component to relapse. After bimaxillary surgery over long observation periods, there was always a greater distorotation of the mandible, ranging from 0.9 degrees of MP-SN after 15 years [9] to 3.77 degrees of MP-SN after 3.5 years [6].

It should be noted that in all studies on the treatment of AOB, the authors found no relapse that returned the negative overbite on incisors. However, due to the many methods of molar intrusion, orthognathic surgery and measurements used by various authors, a clear assessment of the advantage of orthodontic methods using molar intrusion over orthognathic surgery modalities is not possible.

Since the treatment of AOB remains a demanding clinical problem for both orthodontists and maxillofacial surgeons, any attempt to introduce new treatments for this problem becomes extremely valuable, especially if the new treatment method is less invasive and remains at least comparably effective. In orthodontic and surgical treatment of malocclusion, it is extremely important to maintain stable treatment results and prevent complications. As this systematic review aimed to objectively assess the possibility of using skeletal anchorage for molar intrusion in the treatment of AOB as an alternative to surgical orthognathic treatment with regard to the stability of the achieved treatment effects over a long time period, it should also be considered in the discussion on the more common use of less invasive treatments for AOB.

### Limitations

The limitation of the conducted review is the fact that there are no randomized clinical trials objectively evaluating the short- and long-term results of AOB treatment using molar intrusion with skeletal anchorage and the results of AOB treatment with orthognathic surgery. Moreover, some articles that qualified for this review included a very limited number of analyzed cases. The problem with all articles included in the review is the lack of untreated control groups. Randomization is a prerequisite for determining the best treatment options. Moreover, in selected articles, there is a high heterogeneity of results, which makes it impossible to perform a meta-analysis of the results and make firm conclusions.

## 5. Conclusions

It is possible to obtain successful results of AOB treatment in non-growing patients and adults by means of the intrusion of molar teeth with skeletal anchorage and achieve a positive overbite on the incisors, followed by decreases in AFH and CCW rotation of the mandible.

Comparisons of the outcomes of mandibular autorotation as a result of either molar intrusion or orthognathic surgery are extremely difficult due to the heterogeneity of measurements used in research papers.

Due to numerous methods of molar intrusion, different surgical methods applied and different methods of assessing long-term treatment outcomes used by different authors, it is not possible to state conclusively whether the treatment of AOB by means of molar intrusion with skeletal anchorage provides the same long-term results as orthognathic surgery procedures.

## Figures and Tables

**Table 1:** Search terms used to extract suitable articles related to the topic of the review.

Problem	Intervention Q1	Intervention Q2	Outcome
“anterior open bite”	“posterior teeth intrusion”	“orthognathic surgery”	“anterior open bite correction”
AOB	“molar intrusion”	LeFort I	“AOB correction”
Adult *	“absolute anchorage”	LeFort 1	“positive overbite”
Non-growing	“skeletal anchorage”	“bilateral sagittal split osteotomy”	“mandibular autorotation”
Nongrowing	“temporary anchorage”	BSSO	
Adolescent *	TAD		

Q1—molar intrusion with skeletal anchorage; Q2—orthognathic surgery procedures including LIO with or without BSSO mandibular surgery; AOB—anterior open bite; TAD—temporary anchorage device; BSSO—bilateral sagittal split osteotomy; *—any group of characters, including no character.

**Table 2:** Assessment of the risk of bias in the included articles by the methodological index for non-randomized trials tool (MINOR).

	Deguchi et al. 2011
1. A clearly stated aim	2
2. Inclusion of consecutive patients	2
3. Prospective collection of data	2
4. Endpoints appropriate to the aim of the study	2
5. Unbiased assessment of the study end point	0
6. Follow-up period appropriate	2
7. Loss to follow-up less than 5%	2
8. Prospective calculation of the study size	0
9. An adequate control group	2
10. Contemporary groups	2
11. Baseline equivalence of groups	1
12. Adequate statistical analysis	2
TOTAL:	19

**Table 3:** Assessment of the risk of bias in the included articles by the National Institute of Health (NIH) quality assessment tool for cohort studies.

	Scheffler et al. 2014	Marzouk and Kassem 2016	Teittinen et al. 2012	Swinnen et al. 2001	Fischer et al. 2000	Proffit et al. 2000
1. Was the research question or objective in this paper clearly stated?	2	2	2	2	2	2
2. Was the study population clearly specified and defined?	2	1	2	2	1	2
3. Was the participation rate of eligible persons at least 50%?	0	0	0	0	0	0
4. Were all the subjects selected or recruited from the same or similar populations (including the same time period)? Were inclusion and exclusion criteria for being in the study prespecified and applied uniformly to all participants?	1	1	2	1	1	1
5. Was a sample size justification, power description, or variance and effect estimates provided?	0	1	0	1	0	0
6. For the analyses in this paper, were the exposure(s) of interest measured prior to the outcome(s) being measured?	2	2	2	2	2	1
7. Was the timeframe sufficient so that one could reasonably expect to see an association between exposure and outcome if it existed?	2	1	2	1	1	1
8. For exposures that can vary in amount or level, did the study examine different levels of the exposure as related to the outcome (e.g., categories of exposure, or exposure measured as continuous variable)?	0	0	0	0	0	0
9. Were the exposure measures (independent variables) clearly defined, valid, reliable, and implemented consistently across all study participants?	2	1	2	2	2	2
10. Was the exposure(s) assessed more than once over time?	2	2	2	2	2	0
11. Were the outcome measures (dependent variables) clearly defined, valid, reliable, and implemented consistently across all study participants?	2	1	2	2	2	2
12. Were the outcome assessors blinded to the exposure status of participants?	0	0	0	0	0	0
13. Was loss to follow-up after baseline 20% or less?	1	2	1	1	2	2
14. Were key potential confounding variables measured and adjusted statistically for their impact on the relationship between exposure(s) and outcome(s)?	0	0	0	0	0	0
TOTAL:	16	14	17	16	15	13

**Table 4:** Assessment of the risk of bias in the included articles by the Institute of Health Economics (IHE) Appraisal Tool.

	Baek et al. 2010	Sugawara et al. 2002	Ding et al. 2007
1. Is the hypothesis/aim/objective of the study clearly stated?	2	2	2
2. Are the characteristics of the participants included in the study described?	2	1	1
3. Were the cases collected in more than one center?	0	0	0
4. Are the eligibility criteria (i.e., inclusion and exclusion criteria) for entry into the study clearly stated?	2	2	2
5. Were participants recruited consecutively?	1	0	0
6. Did participants enter the study at a similar stage in the disease?	1	1	1
7. Was the intervention of interest clearly described?	2	2	2
8. Were additional interventions (co-interventions) reported in the study?	0	0	0
9. Were the outcome measures established a priori?	2	2	1
10. Were the relevant outcomes measured with appropriate objective and/or subjective methods?	2	2	2
11. Were the relevant outcomes measured before and after the intervention?	2	2	2
12. Were the statistical tests used to assess the relevant outcomes appropriate?	2	2	2
13. Was the length of follow-up reported?	2	2	2
14. Was the loss to follow-up reported?	2	2	2
15. Does the study provide estimates of the random variability in the data analysis of relevant outcomes?	0	0	0
16. Are the adverse events related to the intervention reported?	1	0	1
17. Are the conclusions of the study supported by the results?	2	2	2
18. Are both competing interests and sources of support for the study reported?	2	2	2
TOTAL:	27	24	24

**Table 5:** The strength of evidence for the evaluated outcomes.

							Number of Participants		Effect			
Number of Studies	Study Design (s)	Risk of Bias	Inconsistency	Indirectness	Imprecision	Other Considerations	Intervention	Alternative Intervention	Relative (95% CI)	Absolute (95% CI)	Quality	Importance
The outcomes achieved by molar intrusion with skeletal anchorage												
The change in overbite on the incisors												
5	non-RCT, Coh, CS	moderate	not serious	not serious	not serious	no blinding	77	15		3.2 4.9	moderate	important
The change in lower facial height												
4	non-RCT, Coh, CS	moderate	not serious	not serious	not serious	no blinding, low ss	51	15			moderate	important
Mandibular autorotation												
5	non-RCT, Coh, CS	moderate	not serious	not serious	not serious	no blinding	77	15		−2.2 −1.4	moderate	important
The change in overbite on the incisors (1-year follow-up)												
5	non-RCT, Coh, CS	moderate	not serious	not serious	not serious	no blinding	77	15			moderate	important
The change in lower facial height (1-year follow-up)												
4	non-RCT, Coh, CS	moderate	not serious	not serious	not serious	no blinding, low ss	18	15			low	not important
Mandibular distorotation (1-year follow-up)												
4	non-RCT, Coh, CS	moderate	not serious	not serious	not serious	no blinding, low ss	44	15			moderate	important
The outcomes achieved by orthognathic surgery procedures												
The change in overbite on the incisors												
4	Coh, CS	moderate	not serious	not serious	not serious	no blinding	141			2.4 3.6	moderate	important
Mandibular autorotation												
4	Coh, CS	moderate	not serious	not serious	not serious	no blinding	141			−4.8 −2.2	moderate	important
The change in overbite on the incisors (1-year follow-up)												
2	Coh	moderate	not serious	not serious	not serious	no blinding	107				moderate	important

Non-RCT—non-randomized clinical trial, Coh—cohort studies, CS—case series, ss—sample size, CI—confidence interval.

**Table 6:** Characteristics of studies included in the review.

Study	Type of Intervention	Study Design	Number of Patients	Mean Age of Patients or Range (Years)	Mean Active Treatment Time (Months)	Analyzed Measurements for This Review	Maximum Follow-Up Time (Years)
Baek et al. 2010	Molar intrusion with mini-implants and elastomeric chain and transpalatal bar	Prospective	9	23.7	7.8	Overbite SN-GoMe anterior face height; U6-PP	3
Scheffler et al. 2014	Temporary anchorage devices in the zygomatic buttress area connected to the acrylic splint with NiTi coil springs	Retrospective	33	24.1	6.6	Overbite SN-GoGn lower face height U6-PP	2
Sugawara et al. 2002	Zygomatic mini-plates	Retrospective	9	21.1	14.9	Overbite; MP-FH lower face height U6-PP	1
Deguchi et al. 2011	Miniscrews on the buccal side of molar area with power chain or ligature wire	Prospective, Non-randomized Clinical trial	15	25.7	36	Overbite SN-MP; U6-PP lower face height	2
Marzouk and Kassem 2016	Zygomatic titanium mini-plates fixed with 3 screws	Retrospective	26	22.5	7.5	Overbite SN-MP; U6-PP	3
Ding et al. 2007	Surgical-orthodontic, LefFort I and BSSO; fixation with plates and screws	Retrospective	10	24.5	NA	Overbite SN-MP SN-PP; MP-PP	15
Teittinen et al. 2012	surgical-orthodontic, maxillary or bimaxillary	Retrospective	24 12 maxillary 12 bimaxillary	29.3 (maxillary) 30.8 (bimaxillary)	NA	Overbite SN-MP SN-PP; MP-PP	3
Swinnen et al. 2001	surgical-orthodontic, maxillary or bimaxillary	Retrospective	49	20.9 (women) 20.1 (men)	NA	Overbite SN-PP; N-Me ANS-Me	1
Fischer et al. 2000	surgical-orthodontic, LefFort I and BSSO	Retrospective	58	23	NA	Overbite SN-MP MP-PP	1
Proffit et al. 2000	surgical-orthodontic, maxillary or bimaxillary	Retrospective	54 28 maxillary 26 bimaxillary	21.8 (maxillary) 24.5 (bimaxillary)	NA	Overbite Mandibular plane change Maxillary plane change	3

BSSO—bilateral sagittal split osteotomy; NiTi—nickel–titanium; NA—not applicable; SN-GoMe, U6-PP, SN-GoGn, MP-PP, FMA, U6-HRL, MP-FH, SN-MP, SN-PP, N-Me, ANS-Me—there are according explanations in Table 7.

**Table 7:** Cephalometric measurements used in selected articles relevant to this review.

Measurement	Type of Measurement	Definition of the Measurement
Overbite	Linear	Distance between the incisal edges of the upper central incisor (U1) and the lower central incisor (L1) perpendicular to the horizontal reference line (HRL)
SN-GoMe	Angular	Angle formed by the line going through cephalometric points sella (S)–nasion (N) and the line passing through the points gonion (Go)–menton (Me)
SN-GoGn	Angular	Angle formed by the line passing through cephalometric points sella (S)-nasion (N) and the line passing through the points gonion (Go)–gnathion (Gn)
MP-PP	Angular	Angle formed by the mandibular plane (MP) and the palatal plane (PP)
FMA	Angular	Angle formed by Frankfort horizontal plane and mandibular plane
SN-MP	Angular	Angle formed by the line going through cephalometric points sella (S)–nasion (N) and mandibular plane
SN-PP	Angular	Angle formed by the line going through cephalometric points sella (S)–nasion (N) and palatal plane
MP-FH	Angular	Angle formed by mandibular plane (MP) and Frankfort horizontal plane (FH); synonym of FMA
N-Me	Linear	Distance between nasion (N) and menton (Me)
ANS-Me	Linear	Distance between anterior nasal spine (ANS) and menton (Me)
U6-PP	Liner	Perpendicular distance between mesiobuccal cusp of the upper first molar and palatal plane (PP)
U6-HRL	Linear	Perpendicular distance between mesiobuccal cusp of the upper first molar and horizontal reference line (HRL)
Anterior face height (AFH)	Linear	Distance between nasion (N) and menton (Me)
Lower face height (LFH)	Linear	Distance between anterior nasal spine (ANS) and menton (Me)

**Table 8:** The change in overbite measured on the incisors as a result of anterior open bite treatment by molar intrusion using skeletal anchorage (mm).

Study	Pre-Treatment Mean (SD)	Post-Treatment Mean (SD)	Change in Mean (SD)
Baek et al. 2010	−3.91 (1.65)	1.65 (0.82)	5.56 (1.94) *
Scheffler et al. 2014	−1.2 (1.7)	1.0 (NR)	2.2 (1.6) ^SNR^
Sugawara et al. 2002	−2.8 (1.8)	2.1 (0.8)	4.9 (NR) ^SNR^
Deguchi et al. 2011	−4.4 (1.2)	1.8 (1.1)	6.2 (1.7) *
Marzouk and Kassem 2016	−4.7 (2.3)	2.18 (0.48)	6.93 (1.99) **

NR—not reported; SD—standard deviation; * significant difference compared with pre-treatment (*p* < 0.05); ** significant difference compared with pre-treatment (*p* < 0.01); ^SNR^—significance not reported.

**Table 9:** The change in anterior facial height or lower facial height as a result of anterior open bite treatment by molar intrusion using skeletal anchorage (mm).

Study	Measurement	Pre-Treatment Mean (SD)	Post-Treatment Mean (SD)	Change in Mean (SD)
Baek et al. 2010	AFH	133.95 (5.55)	131.41 (6.10)	−2.53 (1.90)
Scheffler et al. 2014	LFH	NR	NR	−1.6 (2.2)
Sugawara et al. 2002	LFH	76.1 (5.8)	74.6 (6.0)	−1.5 (NR)
Deguchi et al. 2011	LFH	74.7 (5.9)	72.2 (5.1)	−2.6 (2.5)
Marzouk and Kassem 2016	NR	NR	NR	NR

NR—not reported; SD—standard deviation; AFH—anterior facial height; LFH—lower face height.

**Table 10:** The change in overbite measured on the incisors as a result of orthognathic surgery (mm).

Study	Pre-Treatment Mean (SD)	Pre-Surgery Mean (SD)	Post-Surgery Mean (SD)	Change in Mean (SD)
Ding et al. 2007	−3.2 (NR)	−3.2 (NR)	0.6 (NR)	3.8 (NR)
Teittinen et al. 2021	NR NR	−2.55 (1.41) M −2.19 (1.44) B	1.23 (1.05) M 0.98 (1.53) B	3.78 (NR) M 3.17 (NR) B
Swinnen et al. 2001	−0.7 MI −2.1 ME	−0.6 MI −1.9 ME	1.3 MI 0.2 ME	1.9 MI 2.1 ME
Fischer et al. 2000	NR	−0.9 (2.6)	1.3 (1.1)	2.2 (2.4)
Proffit et al. 2000	NR	NR	NR	NR

NR—not reported; SD—standard deviation; M—maxillary group; B—bimaxillary group; MI—maxillary intrusion; ME—maxillary extrusion.

**Table 11:** The change in the facial height as a result of orthognathic surgery (mm).

Study	Measurement	Pre-Treatment Mean (SD)	Pre-Surgery Mean (SD)	Post-Surgery Mean (SD)	Change in Mean (SD)
Swinnen et al. 2001	N-Me	139.1 MI 135.9 ME	139.7 MI 137.1 ME	134.2 MI 136.3 ME	−5.5 MI −0.8 ME
	ANS-Me	139.1 MI 135.9 ME	139.7 MI 137.1 ME	134.2 MI 136.3 ME	−5.5 MI −0.8 ME

MI—maxillary intrusion; ME—maxillary extrusion; N-Me—distance from nasion (N) to menton (Me); ANS-Me—distance from anterior nasal spine (ANS) to menton (Me).

**Table 12:** The results of measurements indicating mandibular autorotation as a result of anterior open bite treatment by molar intrusion using skeletal anchorage (degrees).

Study	Measurement	Pre-Treatment Mean (SD)	Post-Treatment Mean (SD)	Change in Mean (SD)
Baek et al. 2010	SN-GoMe	45.44 (4.11)	43.41 (4.41)	−2.03 (1.59)
Scheffler et al. 2014	SN-GoGn	NR	NR	−1.2 (1.0)
Sugawara et al. 2002	MP-FH	33.1 (2.1)	31.7 (2.4)	−1.3 (NR)
Deguchi et al. 2011	MP-SN	45.8 (6.0)	42.2 (6.7)	−3.6 (2.1)
Marzouk and Kassem 2016	MP-SN	49.1 (3.1)	46.9 (3.9)	−2.13 (0.21)

NR—not reported; SN-GoMe—angle formed by sella–nasion (SN) line and gonion–menton (Go-Me) line; SN-GoGn—angle formed by sella–nasion (SN) line and gonion–gnathion (Go-Gn) line; MP-FH—angle formed by mandibular plane and Frankfort horizontal plane; MP-SN—angle formed by mandibular plane and sella–nasion (SN) line.

**Table 13:** Mean change in measurements indicating mandibular autorotation as a result of orthognathic surgery (degrees).

Study	Measurement	Pre-Treatment Mean (SD)	Pre-Surgery Mean (SD)	Post-Surgery Mean (SD)	Change in Mean (SD)
Ding et al. 2007	PP-SN	11.0 (NR)	11.0 (NR)	15.8 (NR)	4.8 (NR)
	MP-SN	42.0 (NR)	42.5 (NR)	41.2 (NR)	−1.3 (NR)
	MP-PP	31.1 (NR)	31.1 (NR)	25.0 (NR)	−6.1 (NR)
Teittinen et al. 2021	PP-SN	NR	5.15 (2.16) M	9.59 (3.23) M	4.44 (NR) M
			5.49 (3.91) B	8.27 (3.91) B	2.78 (NR) B
	MP-SN	NR	38.15 (6.33) M	34.17 (7.30) M	−3.95 (NR) M
			42.08 (9.27) B	37.48 (8.47) B	−4.6 (NR) B
	MP-PP	NR	32.98 (6.57) M	26.17 (5.78) M	−6.81 (NR) M
			36.57 (9.40) B	29.24 (7.10) B	−7.33 (NR) B
Swinnen et al. 2001	PP-SN	7.9 (NR) MI	7.8 (NR) MI	9.2 (NR) MI	1.4 (NR) MI
		8.9 (NR) ME	9.4 (NR) ME	11.8 (NR) ME	2.4 (NR) ME
Fischer et al. 2000	MP-SN		46.2 (6.8)	42.2 (6.7)	−4.0 (3.1)
	MP-PP		39.6 (6.0)	35.0 (6.6)	−4.6 (4.6)
Proffit et al. 2000	NR	NR	NR	NR	NR

NR—not reported; M—maxillary group; B—bimaxillary group; MI—maxillary intrusion; ME—maxillary extrusion; MP-SN—angle formed by mandibular plane and sella–nasion (SN) line; MP-PP—angle formed by mandibular plane and palatal plane; PP-SN—angle formed by palatal plane and sella–nasion (SN) line.

**Table 14:** Changes in selected values characterizing stability of results of AOB treatment by molar intrusion using skeletal anchorage.

Study	Measurement	Pre-Treatment Mean (SD)	1-Year Follow-Up Mean (SD)	Change in Mean (SD)	2-Year Follow-Up Mean (SD)	Change in Mean (SD)	3-Year Follow-Up Mean (SD)	Change in Mean (SD)
Baek et al. 2010	Overbite	1.65 (0.82)	0.66 (0.79)	−0.99 *	NR	NR	0.45 (1.09)	−0.44 *
	AFH	131.41 (6.10)	131.86 (5.54)	0.45 *	NR	NR	132.32 (5.87)	0.91 *
	SN-GoMe	43.41 (4.41)	43.68 (4.88)	0.29 *	NR	NR	43.98 (4.76)	0.57 *
	U6-PP	24.50 (1.64)	24.89 (1.69)	0.39 *	NR	NR	24.94 (1.68)	0.44 *
Scheffler et al. 2014	Overbite	1.0 (NR)	0.7 (NR)	−0.3 *	0.3 (NR)	−0.7 *	NR	NR
	LFH	NR	NR	0.2 (1.4)	NR	0.3 (1.4)	NR	NR
	SN-GoGn	NR	NR	0.0 (NR)	NR	0.0 (NR)	NR	NR
	U6-PP	NR	NR	0.5 (1.1)	NR	1.0 * (1.1)	NR	NR
Sugawara et al. 2002	Overbite	2.1 (0.8)	1.2 (0.8)	−0.9 *	NR	NR	NR	NR
	LFH	74.6 (6.0)	75.2 (5.8)	0.6 *	NR	NR	NR	NR
	MP-FH	31.7 (2.4)	32.2 (3.0)	0.5 *	NR	NR	NR	NR
	U6-PP	25.0 (2.8)	25.1 (2.5)	0.1 *	NR	NR	NR	NR
Deguchi et al. 2011	Overbite	1.8 (1.1)	1.0 (0.9)	−0.8	NR	NR	NR	NR
	LFH	72.2 (5.1)	72.2 (5.1)	0.0 *	NR	NR	NR	NR
	MP-SN	42.2 (6.7)	43.8 (6.5)	1.6 *	NR	NR	NR	NR
	U6-PP	24.6 (2.5)	25.1 (2.8)	0.5 *	NR	NR	NR	NR
Marzouk and Kassem 2016	Overbite	2.18 (0.48)	1.61 (0.42)	−0.57 *	NR	NR	1.41 (0.39)	−0.2 *
	MP-SN	46.9 (3.9)	47.2 (3.9)	0.3 *	NR	NR	47.4 (3.9)	0.2 *
	U6-PP	25.23 (2.14)	25.54 (2.17)	0.31	NR	NR	25.64 (2.17)	0.10 *

*—calculated value; NR—not reported; SD—standard deviation; AFH—anterior face height; LFH—lower face height; SN-GoMe—angle formed by sella–nasion (SN) line and gonion–menton (Go-Me) line; SN-GoGn—angle formed by sella–nasion (SN) line and gonion–gnathion (Go-Gn) line; FMA—angle formed by Frankfort horizontal line and mandibular plane; MP-FH—angle formed by mandibular plane and Frankfort horizontal plane; MP-SN—angle formed by mandibular plane and sella–nasion (SN) line.

**Table 15:** Changes in selected values characterizing stability of results of AOB treatment by orthognathic surgery procedures.

Study	Observation Time (Years)	Measurement	Post-Surgery Mean (SD)	Follow-Up Mean (SD)	Change in Mean (SD)
Ding et al. 2007	15	Overbite	0.6 (NR)	1.5 (NR)	0.9 (NR)
		PP-SN	15.8 (NR)	13 (NR)	−2.6 (NR)
		MP-SN	41.2 (NR)	42.1 (NR)	0.9 (NR)
		MP-PP	25.0 (NR)	28 (NR)	2.9 (NR)
Teittinen et al. 2021	3.5	Overbite	1.23 (1.05) M	1.85 (0.93) M	0.62 * M
			0.98 (1.53) B	0.73 (0.93) B	−0.25 B
		PP-SN	9.59 (3.23) M	7.45 (3.08) M	−2.14 M
			8.27 (3.91) B	7.06 (4.14) B	−1.21 B
		MP-SN	34.17 (7.30) M	35.84 (5.95) M	1.67 M
			37.48 (8.47) B	41.25 (10.37) M	3.77 B
		MP-PP	26.17 (5.78) M	28.38 (5.80) B	2.21 M
			29.24 (7.10) B	34.20 (8.78) M	4.96 B
Swinnen et al. 2001	1	Overbite	1.3 (NR) MI	1.8 (NR) MI	0.5 * MI
			0.2 (NR) ME	0.8 (NR) ME	0.6 * ME
		PP-NS	9.2 (NR) MI	8.0 (NR) MI	−1.2 * MI
			11.8 (NR) ME	9.3 (NR) ME	−2.5 * ME
		N-Me	134.2 (NR) MI	133.6 (NR) MI	−0.6 * MI
			136.3 (NR) ME	134.2 (NR) ME	−2.1 * ME
		ANS-Me	81.3 (NR) MI	81.7 (NR) MI	0.4 * MI
			76.3 (NR) ME	75.4 (NR) ME	−0.9 * ME
Fischer et al. 2000	1	Overbite	1.3 (2.6)	0.8 (1.4)	−0.5 (1.3)
		MP-SN	42.2 (6.7)	43.7 (6.7)	1.4 (2.0)
		MP-PP	35.0 (6.6)	36.7 (6.3)	1.7 (2.8)
Proffit et al. 2000	3	Overbite	NR	NR	0.02 (1.21) M
			NR	NR	−0.25 (1.25) B

*—calculated value; NR—not reported; SD—standard deviation; MP-SN—angle formed by mandibular plane (MP) and sella–nasion (SN) line; PP-SN—angle formed by palatal plane (PP) and sella–nasion (SN) line; MP-PP—angle formed by mandibular plane (MP) and palatal plane (PP); N-Me—distance between nasion (N) and menton (Me); ANS-Me—distance between anterior nasal spine (ANS) and menton (Me), overbite, N-Me, ANS-Me expressed in mm; PP-SN, MP-SN, MP-PP expressed in degrees.
